# Omega‐3 multiple effects increasing glucocorticoid‐induced muscle atrophy: autophagic, AMPK and UPS mechanisms

**DOI:** 10.14814/phy2.13966

**Published:** 2019-01-15

**Authors:** Alan Fappi, Juliana de C. Neves, Karine A. Kawasaki, Luana Bacelar, Leandro N. Sanches, Felipe P. da Silva, Rubens Larina‐Neto, Gerson Chadi, Edmar Zanoteli

**Affiliations:** ^1^ Department of Neurology Faculdade de Medicina FMUSP Universidade de Sao Paulo SP, Brazil

**Keywords:** autophagy, eicosapentaenoic acid, glucocorticoid, IGF‐1 pathway, MEK/ERK pathway, muscle atrophy, Myostatin/Smad2/3 pathway, omega‐3 fatty acid

## Abstract

Muscle atrophy occurs in many conditions, including use of glucocorticoids. N‐3 (omega‐3) is widely consumed due its healthy properties; however, concomitant use with glucocorticoids can increase its side effects. We evaluated the influences of N‐3 on glucocorticoid atrophy considering IGF‐1, Myostatin, MEK/ERK, AMPK pathways besides the ubiquitin‐proteasome system (UPS) and autophagic/lysosomal systems. Sixty animals constituted six groups: CT, N‐3 (EPA 100 mg/kg/day for 40 days), DEXA 1.25 (DEXA 1.25 mg/kg/day for 10 days), DEXA 1.25 + N3 (EPA for 40 days + DEXA 1.25 mg/kg/day for the last 10 days), DEXA 2.5 (DEXA 2.5 mg/kg/day for 10 days), and DEXA 2.5 + N3 (EPA for 40 days + DEXA 2.5 mg/kg/day for 10 days). Results: N‐3 associated with DEXA increases atrophy (fibers 1 and 2A), FOXO3a, P‐SMAD2/3, Atrogin‐1/MAFbx (mRNA) expression, and autophagic protein markers (LC3II, LC3II/LC3I, LAMP‐1 and acid phosphatase). Additionally, N‐3 supplementation alone decreased P‐FOXO3a, PGC1‐alpha, and type 1 muscle fiber area. Conclusion: N‐3 supplementation increases muscle atrophy caused by DEXA in an autophagic, AMPK and UPS process.

## Introduction

Muscle mass maintenance depends essentially on the balance between protein synthesis and degradation. Deregulation of these regulatory processes results in a trophic response, either muscle hypertrophy or atrophy (Nader 2005; Matsakas and Patel 2009). Muscle atrophy corresponds to the shrinkage of myofibers due to the loss of protein networks, organelles, and cytoplasm owing especially to increased expression of atrogenes and inhibition of the proteins that regulate muscle mass synthesis and trophism (i.e., Akt/mTOR/P70S6K) (Glass 2003; Boonyarom and Inui 2006; Schiaffino et al. 2013). This catabolic condition may occur in response to physiological adaptations to fasting, inactivity, and aging; pathological conditions such as septicemia, diabetes, cachexia, AIDS, cancer, spinal cord injury, myopathies, neuropathies, and other genetically inherited diseases; or prolongated intake of exogenous glucocorticoids (Voisin et al. 1996; Glass 2003; Kandarian and Jackman 2006; Pereira and Freire de Carvalho 2011).

Synthetic glucocorticoids represent one of the most prescribed therapeutic compounds and are widely used in the treatment of inflammatory, autoimmune, and proliferative lymphocytic diseases (Nicolaides et al. 2014). They are also a very important complement treatments for Duchenne's muscle dystrophy (Bushby et al. 2010; Nicolaides et al. 2010). On the other hand, glucocorticoids can lead to important side effects in the skeletal muscle system and cause the most common drug‐induced myopathy. Type 2B/2X muscle fibers are the most affected, while little or no effect is observed on other fiber types (Schacke et al. 2002; Gupta and Gupta 2013). This higher susceptibility of type 2 fibers to glucocorticoids seems to be related to a lower level of PGC‐1*α* in this fiber type (Sandri et al. 2006).

Glucocorticoid‐induced muscle atrophy comprises IGF‐1 (insulin‐like growth factor) pathway suppression and myostatin pathway activation (Schakman et al. 2013). Thus, FoxO, mTOR, Akt, GSK‐3*β*, and other transcription factors are expected to become therapeutic targets to promote direct inhibition of one important protein breakdown mechanism related to glucocorticoid‐induced muscle atrophy, the ubiquitin‐proteasome system (UPS). Some candidates for glucocorticoid muscular atrophy attenuation are testosterone (Qin et al. 2010; Wu et al. 2010), creatine (Menezes et al. 2007), and HMB leucine metabolite (Aversa et al. 2012).

Major omega‐3 (N‐3) fatty acids are eicosapentaenoic acid (EPA) (C20: 5 n‐3), docosahexaenoic acid (DHA) (C22: 6 n‐3), and *α*‐linolenic acid (ALA) (C18: 3 n‐3), which are present especially in marine vegetables and animals such as salmon (0.84 and 0.81 g/100 g), sardines (0.47 and 0.51 g/100 g), caviar (1.03 and 1.35 g/100 g), and oyster (0.42 and 0.46 g/100 g) (Kris‐Etherton et al. 2002). N‐3 consumption promotes innumerable benefits to the organism, mostly to the memory and cardiovascular systems (Daviglus et al. 1997; Su 2010). Cognitive benefits are usually associated to DHA, and cardiovascular benefits may occur in response to EPA and ALA (Muldoon et al. 2010). There is still no consensus concerning the ideal daily dose of EPA/DHA (Vannice and Rasmussen 2014). According to the U.S. National Institutes of Health (NIH), fish oil is one of the nonvitamin/nonmineral dietary supplements most commonly used by U.S. adults and children, and the recommended N‐3 daily intake is 1.6 g/day for males and 1.1 g/day for females (National Institutes of Health, 2018).

Previous studies have shown that N‐3 supplementation can be helpful in cancer‐associated muscle atrophy and septicemia, reducing the activity of UPS components and NF‐kB pathway modulation (Whitehouse et al. 2001; Tisdale 2007; Khal and Tisdale 2008). Curiously, our previous study indicated a possible aggravation of glucocorticoid‐induced muscle atrophy when a combination of N‐3 (100 mg/kg/day) and dexamethasone (DEXA) was administrated to rats (Fappi et al. 2014). Animals treated with glucocorticoids and N‐3 developed atrophy in type 1 and 2A fibers and had an increased *Atrogin‐1* mRNA expression compared to those receiving only dexamethasone. In the present study, we aimed to better elucidate the mechanisms of skeletal muscle atrophy due to combined administration of N‐3 and dexamethasone in different dosages.

## Materials and Methods

### Animals and drug treatment

Male Wistar rats (60 animals) aged between 10 and 12 weeks and weighing between 320 and 350 g were used. Animals were housed in cages containing no more than three individuals under dark‐light cycles of 12 h each at 25°C and received food and water *ad libitum*. The animals received commercial feed (Nuvital, Nuvilab CR‐1) containing crude protein (min 22.0%), ethereal extract (min 4.5%), mineral matter (max 1 d0.0%), fibrous matter (max 8.0%), calcium (max 1.4%), phosphor (min 0.8%), vitamin A 25,200.00 UI/kg, vitamin D3 2,100.00 UI/kg, vitamin E 60.00 mg/kg, vitamin K3 12.50 mg/kg, vitamin B1 14.40 mg/kg, vitamin B2 11.00 mg/kg, vitamin B6 12.00 mg/kg, vitamin B12 60.00 mcg/kg, niacin 60.00 mg/kg, pantothenic acid 112.00 mg/kg, folic acid 6.00 mg/kg, biotin 0.26 mg/kg, colin 1,100.00 mg/kg, iron 50.00 mg/kg, zinc 60.00 mg/kg, copper 10.00 mg/kg, iodine 2.00 mg/kg, manganese 60.00 mg/kg, selenium 0.05 mg/kg, cobalt 1.50 mg/kg, lysine 100.00 mg/kg, methionine 300.00 mg/kg, and antioxidant 100.00 mg/kg. All *in vivo* experiments were approved by our local research ethics committee (CEUA FMUSP, process 430/2013) and performed according to the NIH guidelines on care, handling, and use of laboratory animals.

### N‐3 and DEXA administration

Thirty animals were supplemented with 100 mg/kg/day of EPA and 20 mg/kg/day of DHA (*Supra* Omega™, Global Nutrition) dissolved in 0.5% Tween 20 in ultrapure water via gavage (v.g.) for 30 days. The remaining 30 animals received only vehicle solution.

In sequence, the animals treated or not with EPA/DHA were equally divided into six experimental groups, and subcutaneous (s.c.) injections of dexamethasone (DEXA, Achè Laboratories, Decadron 4 mg/mL) or saline solution were administered for 10 days as follows: (1) control group (CT): vehicle (v.g.) for 40 days associated with saline solution (s.c.) on the last 10 days, (2) DX1.25 group: vehicle solution (v.g.) for 40 days associated with DEXA 1.25 mg/kg/day on the last 10 days, (3) DX1.25+N‐3: N‐3 supplementation (v.g.) for 40 days associated with DEXA 1.25 mg/kg/day on the last 10 days, (4) DX2.5: vehicle solution (v.g.) for 40 days associated with DEXA 2.5 mg/kg/day on the last 10 days, (5) DX2.5+N‐3: N‐3 supplementation (v.g.) for 40 days associated with DEXA 2.5 mg/kg/day on the last 10 days. The injections were applied in the dorsal region, always alternating sides to prevent wounds.

On the 40th day, the animals were euthanized with sodium pentobarbital intraperitoneal injection (30 mg/kg). Their gastrocnemius (GA) muscles, tibialis anterior (TA) muscles, and adrenal glands (AGs) were immediately dissected, weighed, snap frozen in isopentane, cooled in liquid nitrogen and stored at −80°C.

### Histological analysis

TA cross‐sections were performed in cryostat (Leica, CM3000) at −25°C. The sections were submitted to the metachromatic dye‐ATPase method (mATPase) according to Ogilvie and Feeback (Ogilvie and Feeback 1990) to differentiate the muscle fiber subtypes 1, 2A, and 2B. The slides were photographed at 20X (Olympus, microscope AX70, camera and software Olympus DP72), and the cross‐sectional areas (CSA) were measured for each muscle fiber subtype using analysis tool in Photoshop CS6 extended software. Prior to the measurements, a pixel‐to‐micrometer conversion scale was established in Image J software based on pictures of a micrometer slide at the same magnification. An average of 350 fibers of each muscle fragment was measured.

### Immunohistochemistry

Slides were incubated in 1% triton‐X in PBS for 10 min and washed with 0.02% tween‐20 in PBS and 0.02% tween‐20, 1% BSA in PBS. Then, they were blocked in 2% normal goat serum, 4% BSA, and 0.9% triton‐x in PBS. Anti‐PGC1*α* (Abcam, ab54481) [1:200] was incubated overnight at 4°C and rabbit secondary biotinylated antibody (Vector Laboratories, #BA‐1000) [1:200] for 45 min at room temperature in blocking solution (0.2% triton‐x, 1% normal goat serum, 2% BSA in PBS). After washing in 1% tween‐20 in PBS, endogenous peroxidase activity was quenched with 0.1% H_2_O_2_ in PBS for 30 min, followed by streptavidin incubation for 45 min and washing with 50 mmol/L Trizma in PBS pH7.4. The slides were exposed to the chromogen diaminobenzidine (1.3 mmol/L and 0.05% of H_2_O_2_ in PBS) for 2 min and counterstained with hematoxylin. Subsequently, the slides were dehydrated in ethanol (70–100%), cleared in xylene for 5 min, and mounted with Entellan (Merck 107960). The slides were photographed at 20X magnification for qualitative examination.

### SDS‐PAGE (Western blotting)

TA fragments were homogenized with cooled RIPA buffer (PBS pH7.4, 0.5% sodium deoxycholate, 0.1% SDS, 1 mmol/L EDTA pH8.0,1 mmol/L EGTA pH8.0, 50 mmol/L Tris‐Hcl, 1% NP‐40, 10 mmol/L NaOV, 10 mmol/L NaPyr, 50 mmol/L NaF, and 1% protease inhibitor Sigma P8340) in 20x w/v and centrifuged for 5 min at 4°C at 16,100 g, and the supernatants were quantified using Bradford reagent (Bio‐Rad, #500‐0006) and BSA standard curve. The samples were boiled at 95°C for 5 min and then applied to 8 or 10% bis‐acrylamide mini‐gels, with 50–80 *μ*g protein load per well. In sequence, the samples were transferred to PVDF or nitrocellulose membranes at 65 V for 1 h in a Criterion Blotter (Bio‐Rad, Hercules, CA, USA) apparatus. The membranes were blocked in 5% BSA for 1 h and incubated overnight with primary antibody [1:1000] diluted in blocking solution. Rabbit secondary HRP conjugated antibody (GE, #NA934) [1: 10,000] diluted in blocking solution (5% BSA in TBS‐T) was incubated 1h at room temperature and then ECL (Merck/Millipore, #WBKLS0500) was incubated for 5 min, prior to scanning in C‐DiGit Blot Scanner (LI‐COR) for 12 min. For protein loading control, labeling densities were normalized against GAPDH (glyceraldehyde 3‐phosphate dehydrogenase) of the correspondent sample. Blots were analyzed with Image Studio software version 4.0 (LI‐COR).

The primary antibodies used included anti‐Akt pan (Cell Signaling, #4691), anti‐P‐Akt (Ser473) (Cell Signaling, #4060), anti‐GSK‐3*β* (Cell Signaling, #9315), anti‐P‐GSK‐3*β* (Ser9) (Cell Signaling, #9322), anti‐FOXO3a (Cell Signaling, #2497), anti‐P‐FOXO3a (Ser253) (Cell Signaling, #9466), anti‐ERK1/2 (Cell Signaling, #4695), anti‐P‐ERK1/2 (Thr202/Tyr204) (Cell Signaling, #4377), anti‐Smad2/3 (Cell Signaling, #3102), anti‐P‐Smad2/3 (Ser423/425)/(Ser465/467) (Cell Signaling, #8828), anti‐PGC1*α* (Abcam, #ab54481), anti‐LC3B (Sigma Aldrich, #L7543), and anti‐LAMP1 (Hybridoma Bank, #1D4B).

### Quantitative PCR

Total RNA was extracted from 30mg of GA muscles using SV Total RNA Isolation System (Promega, #Z3105) according to the guidance for Preparation of Lysates from Small Tissue Samples. RNA pellets were resuspended in 50 *μ*L nuclease‐free water for a final RNA concentration of 500 ng/*μ*L. RNA purity and integrity were tested by spectrophotometry and agarose gel, respectively. In sequence, reverse transcription reactions were performed using the GoScript Reverse Transcription Mix Kit (Promega, #A2801) following the manufacturer's instructions. Quantitative PCR was done in duplicate with GoTaq qPCR Master Mix (Promega, #A6002) loading [75ng] of cDNA and [50 nmol/L] of sense and antisense primers in Piko Real 96 (Thermo Scientific, TCR0096) equipment. The data obtained were systematized and analyzed according to the calculation of 2^−∆∆CT^.

Primer sequences: ***MURF‐1*** forward *TCGACATCTACAAGCAGGAA*, reverse *CTGTCCTTGGAAGATGCTTT*;***Atrogin‐1/MAFbx*** forward *TGAAGACCGGCTACTGTGGAAGAGAC*, reverse *TTGGGGTGAAAGTGAGACGGAGCAG*;***REDD‐1*** forward *CACCGGCTTCAGAGTCATCA*, reverse *CGGGTCTCCACCACAGAAAT*;***REDD‐2*** forward *CTTCAGCGTCTGGTGAAATCC*, reverse *ATGCTGGCCGTGTTCTTACTG*;** IRS‐1** forward CCCGGTCGGTGCCAAATAGC, reverse GCCACTGGTGAGGTATCCACATAGC; ***IRS‐2*** forward CCACACACCTGTCCTCATTG, reverse TAATCCGCTTTGCCAAAATC; ***GAPDH*** **(housekeeping gene)** forward *ACGCCAGTAGACTCCACGAC*, reverse *ATGACTCTACCCACGGCAAG*.

### Statistical analysis

The quantitative results were analyzed in GraphPad Prism 5.0 software (GraphPad Software). They were initially classified according to normality using the Shapiro‐Wilk normality test and then evaluated with the appropriate statistical tests. Differences between means were analyzed using the unpaired Student's *t*‐test, and differences among groups were analyzed by one‐way ANOVA followed by Bonferroni post hoc test. The test used and number of individuals per group are described in the presentation of each result. Variations with *P ≤ *0.05 were considered statistically significance.

## Results

### N‐3 supplementation causes a negative influence in cross‐sectional areas (CSA) of type 1 and 2A muscle fibers and affects fast‐to‐slow muscle fiber conversion during DEXA‐induced muscle atrophy

**Figure 1:**
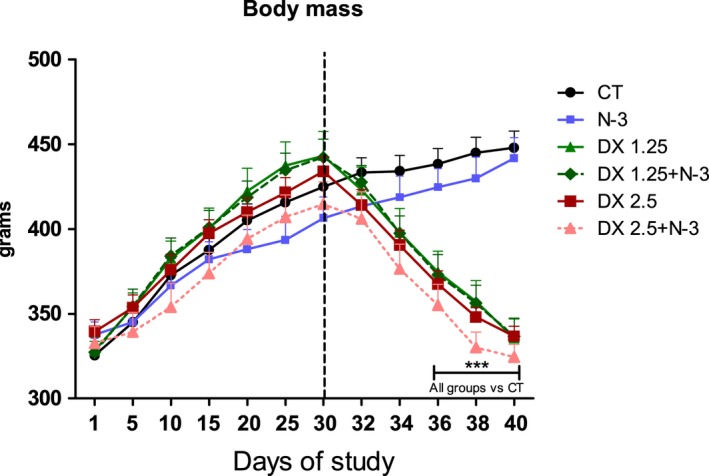
Body mass during the 40 days of the study. The dotted line represents the initial moment of DEXA administration. Data are presented as mean ± SD. Asterisks represent the statistical analysis in comparison to the CT; ***P < 0.001 (*n* per group: CT **= 10, N3* *= 10, DX1.25* *= 9, DX1.25+N3* *= 8, DX2.5* *= 8, DX2.5+N3* *= 9). A repeated‐measures ANOVA followed by Bonferroni post hoc test was used.

Initial body weight between groups ranged from 325.5 to 339.4 g with linear body weight gain proportional to the natural growth of the animals during the initial 30 days of the study (administration of N‐3 or vehicle solution via gavage). Significant body weight loss started 4 days after DEXA administration (2.5 mg/kg/day with or without N‐3) in comparison to the CT group (Fig. 1). From the 6th to the 10th day, DEXA administration caused significant loss of body mass in comparison to the CT group (*P *< 0.01 to all comparisons) without N‐3 influence. The body weight loss after 10 days of DEXA adminstration was about 24% (DX2.5–22.4%, DX1.25–24.3%, DX2.5+N‐3–21.7%, and DX1.25+N‐3–23.8%). The body weight gain was 30.86% in the N‐3 group and 37.60% in the CT group on the 40th day of the study.

To evaluate how much DEXA and N‐3 could directly influence peripheral muscles and adrenal gland mass (indirect evaluation of HPA axis response), the tissues were weighed immediately after collection. The adrenal glands and their respective weights (Table**** 1) showed that DEXA administration at different dosages resulted in significant adrenal weight loss in comparison to the CT group: DX 1.25 (−68.83%), DX 2.5 (−66.91%), DX 1.25+N‐3 (−64.34%), and DX 2.5 + N‐3 (−60.90%). N‐3 in concomitance with DEXA did not aggravate the adrenal weight loss observed in the DX groups.

**Table 1:** Weight of collected adrenal and muscle tissues

	Tissue collected		
	Adrenal	GA	TA
CT	0.063 ± 0.04	2.42 ± 0.21	0.70 ± 0.06
N‐3	0.051 ± 0.01	2.42 ± 0.18	0.66 ± 0.05
DX 1.25	0.019 ± 0.002***	1.61 ± 0.25***	0.51 ± 0.03***
DX 2.5	0.020 ± 0.006**	1.77 ± 0.27***	0.47 ± 0.06***
DX 1.25 + N‐3	0.022 ± 0.008***	1.53 ± 0.13***	0.50 ± 0.05***
DX 2.5 + N‐3	0.024 ± 0.007**	1.80 ± 0.41***	0.50 ± 0.09***

A one‐way ANOVA followed by a Bonferroni post‐test was used (*n* per group: CT* *= 10, N3* *= 10, DX1.25* *= 9, DX1.25+N3* *= 8, DX2.5* *= 8, DX2.5+N3* *= 9).

Asterisks represent the statistical analysis in comparison to the CT.

**P<0.01 and ***P < 0.001.

GA and TA muscle weights significantly decreased in all the groups receiving DEXA associated or not with N‐3, a 30.5% decrease in GA and 28.5% in TA muscle on average. N‐3 did not cause any effect on muscle mass in relation to the CT group or among drugs.

**Figure 2:**
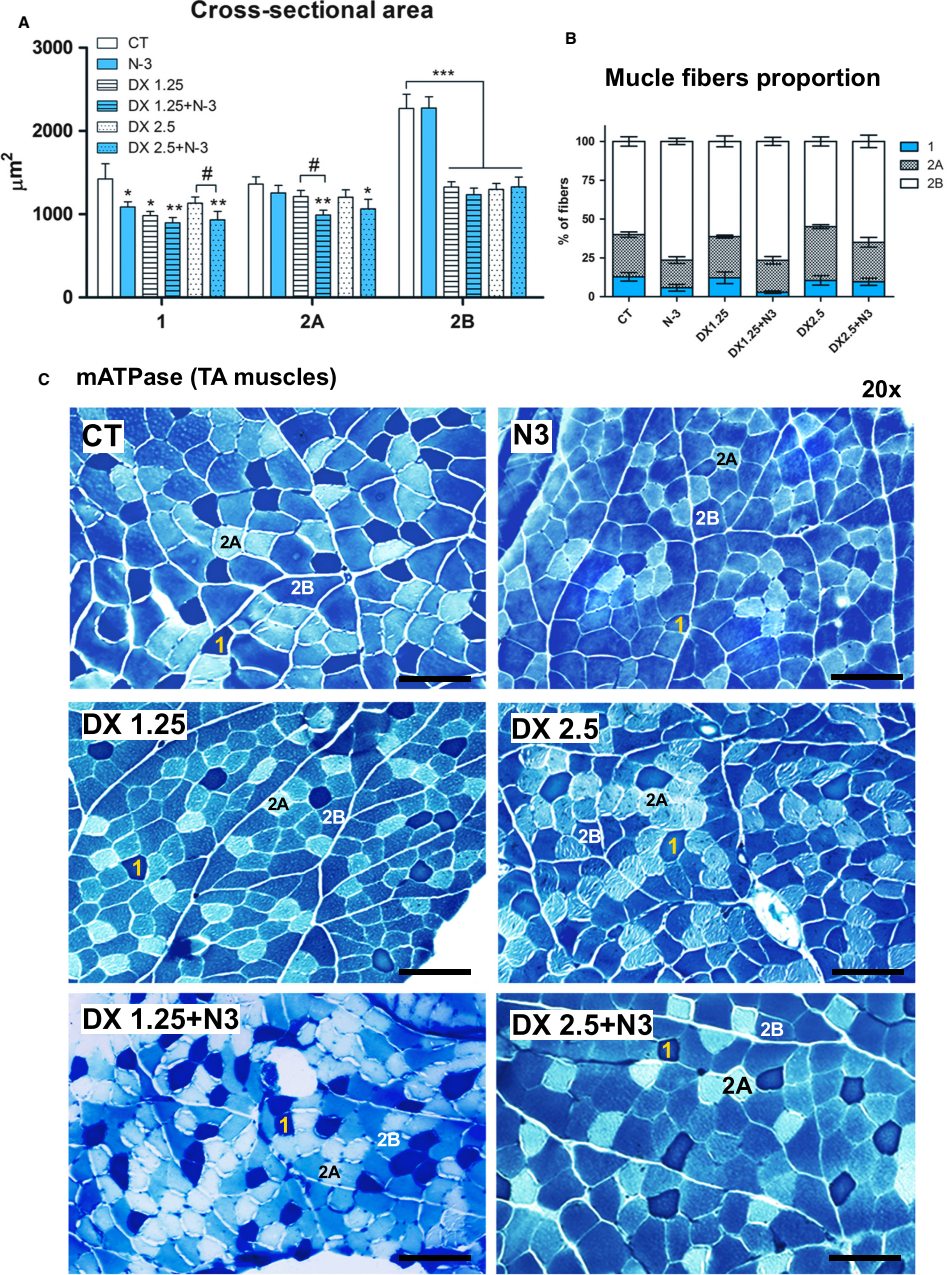
Muscle cross‐sectional area and muscle fiber type proportions analyzed after 40 days of N‐3 supplementation associated or not with dexamethasone on the last 10 days. (A) Cross‐sectional area – (*) represents statistical analysis in comparison to the control group (CT), and (#) represents differences between two groups. Legend: * or # = *P *< 0.05; **=*P *< 0.01 and ***=*P *< 0.001 (*n* per group: CT **= 9, N3* *= 9, DX1.25* *= 9, DX1.25+N3* *= 8, DX2.5* *= 8, DX2.5+N3* *= 7). One‐way ANOVA followed by Bonferroni post hoc test was used for each fiber type analysis; (B) muscle fiber type proportions. Statistical analysis is expressed in Table 2; (C) histological mATPase photographs from all groups.

We used the mATPase method, which differentiates muscle fibers into types 1, 2A, and 2B, to evaluate whether N‐3 could influence the CSA after 10 days of different dosages of DEXA administration (Fig. 2A and B).

There was significant CSA reduction in 2B muscle fibers after DEXA administration associated or not with N‐3 compared to the CT group (*P* < 0.001 for all comparisons); an average decrease of 42% (DX2.5‐44.92%; DX 1.25‐41.63%; DX 2.5+N‐3‐41.46% and DX1.25+N‐3‐45.58%) in 2B fiber CSA compared to the CT group. N‐3 alone or associated to DEXA did not have an influence on type 2B muscle fiber.

With respect to 2A muscle fibers, only the DX+N‐3 groups showed significant CSA reduction in comparison to the CT group (DX1.25+N‐3 – *P *< 0.01 and DX2.5+N‐3 – *P *< 0.05). The decrease in the DX 2.5+N‐3 group was 21.76% and in the DX1.25+N‐3 group was 27.15% in comparison to the CT group. There was a significant decrease in 2A fiber CSA in the DX1.25+N‐3 in comparison to the DX1.25 group (*P *< 0.05).

Type 1 muscle fibers of all groups except the DX2.5 group presented significant CSA reduction in comparison to the CT group, including animals that received only N‐3. CSA reduction in animals in the DX2.5+N‐3 group was higher than that observed in DX2.5 (*P* < 0.05). The decrease in the N‐3 group was 23.65% (*P *< 0.05), in DX1.25 was 30.95 (*P *< 0.05), in DX 1.25+N‐3 was 36.90% (*P *< 0.01), in DX 2.5 was 20.54%, and in DX2.5+N‐3 was 34.52% (*P *< 0.01) compared to the CT group.

Therefore, N‐3 supplementation prior to and concomitant with the DEXA administration had a negative influence on type 1 and 2A muscle fibers CSA.

To identify whether N‐3 could influence the muscle fiber switching during the DEXA‐induced muscle atrophy process, the proportion of muscle fiber types was classified from a pool of 350 muscle fibers per animal (*n* = 5 per group)(Fig. 2C, Table 2).

**Table 2:** Muscle fiber type proportion analysis after 40 days of N‐3 supplementation associated or not with dexamethasone on the last 10 days. The Mann‐Whitney test was used (*n *= 5 per group)

Muscle fiber proportion						
	Type 1	*P* value	Type 2A	*P* value	Type 2B	*P* value
CT	12,83%	*P* = 0.0043** vs DX1.25+N‐3	27,17%	*P* = 0.0087** vs N‐3 *P* = 0.0260* vs DX1.25+N‐3 *P* = 0.0023** vs DX2.5	60,00%	*P* = 0.0022** vs N‐3 *P* = 0.0043** vs DX1.25+N‐3
N‐3	5,77%	‐	17,79%	‐	76,44%	‐
DX1.25	12,18%	‐	26,50%	*P* = 0.0006** vs DX2.5	61,32%	‐
DX1.25+N‐3	2,90%	*P* = 0.0152* vs DX1.25	20,57%	*P* = 0.0411* vs DX1.25	76,53%	*P* = 0.0043** vs DX1.25
DX2.5	10,52%	‐	34,56%	‐	54,92%	‐
DX2.5+N‐3	9,63%	‐	25,38%	*P *= 0.0087** vs *DX2.5*	64,99%	‐

The proportion of type 1 muscle fibers was significantly decreased in the DX1.25+N‐3 group (*P *< 0.05), with a tendency to decrease in the N‐3 group (*P *= 0.08) compared to the CT group. There was also a significant decrease in the proportion of type 1 fibers in the group DX1.25+N‐3 compared to the DX1.25 group (*P *< 0.05).

There was a significant decrease in the proportion of 2A muscle fibers in the N‐3 group (*P *< 0.01) and DX1.25 group (*P *< 0.05) and an increase in the DX2.5 group (*P *< 0.01) compared to the CT group. Significant differences in the proportion of 2A muscle fibers were observed between the DX2.5 and DX 2,5+N‐3 groups (*P *< 0.01) and between the DX1.25 and DX1.25 + N‐3 (*P *< 0.05) groups.

The N‐3 and DX1.25+N3 groups had significantly increased proportions of 2B muscle fibers compared to the CT group (*P *< 0.01 for both). The same was observed when comparing the DX1.25+N‐3 group and DX1.25 group (*P *< 0.01).

In conclusion, DEXA did not have a major influence on the fiber type switch (slow – fast), but N‐3 supplementation prior and concomitant with DEXA could significantly influence the fast‐to‐slow muscle fibers switch during the atrophy process.

### N‐3 decreased P‐FOXO3a and increased atrogene expression when associated with DEXA

**Figure 3:**
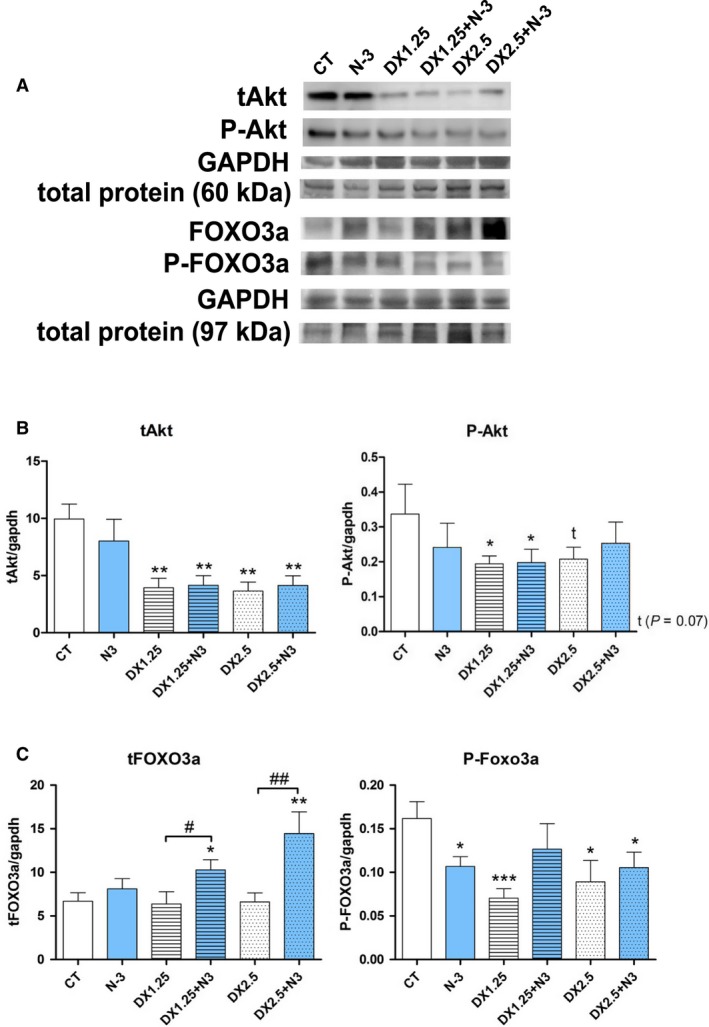
Western blotting analysis of IGF‐1 pathway after 40 days of N‐3 supplementation associated or not with dexamethasone on the last 10 days. (*) represents the statistical analysis in comparison to the CT, and (#) represents differences between groups. (A) representative blots; (B and C) total and phosphorylated Akt and FoxO3a expressions, respectively. Student's *t*‐test was used; Legend: * or #*P *< 0.05; ** or ##*P *< 0.01 and ***P < 0.001 (*n* per group: CT **= 9, N3* *= 10, DX1.25* *= 8, DX1.25+N3* *= 8, DX2.5* *= 8, DX2.5+N3* *= 8).

**Figure 4:**
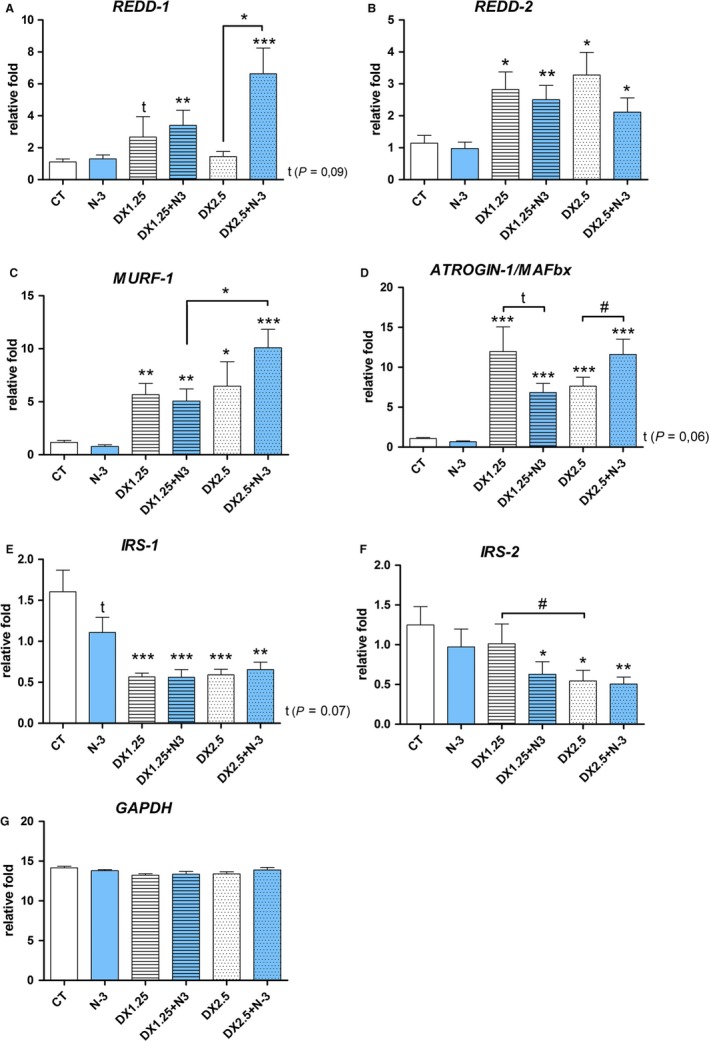
mRNA expression by RT‐PCR of *MURF‐1*,* Atrogin*‐*1/MAFbx*,*REDD‐1*,*REDD‐2*, and *GAPDH* after 40 days of N‐3 supplementation associated or not with dexamethasone on the last 10 days. (*) represents the statistical analysis in comparison to the CT, and (#) represents differences between groups. Student's *t*‐test was used; Legend: * or #*P *< 0.05; ** or ##*P *< 0.01 and *** or ###*P *< 0.001, (*n* per group: CT **= 10, N3* *= 10, DX1.25* *= 9, DX1.25+N3* *= 8, DX2.5* *= 8, DX2.5 + N3* *= 9).

Considering that the muscle atrophy process is directly related to the IGF‐1 pathway, the main components on total and phosphorylated forms (Akt and FoxO3a) protein expression were analyzed by western blotting technique (Fig. 3), and atrogenes *Atrogin‐1/MAFbx, MuRF‐1, REDD‐1 and REDD‐2* (related to the FOXO transcriptional activity) and insulin receptor substracts (IRS‐1, IRS‐2; related to the downstream of IGF‐1 and Insulin receptors) were analyzed by quantitative PCR (Fig. 4).

The total Akt (tAkt) expression was a marked decrease in animals receiving DEXA associated or not with N‐3 compared to the CT group (*P *< 0.01 to all comparison). The N‐3 supplementation did not influence its expression.

The expression of the phosphorylated form of Akt (P‐Akt‐Ser473) was significantly decreased in animals in the DX1.25 and DX1.25+N3 groups (*P *< 0.05 for both) and showed a tendency to decrease in the DX2.5 group (*P *= 0.07) compared to the CT group.

FoxO3a expression, a transcription factor related to E3‐ligase (Atrogin‐1/MAFbx and MuRF‐1) transcription, was significantly increased only in animals receiving DEXA+N‐3 (groups DX1.25+N3 and DX2.5+N3) in relation to the CT group and additionally in comparison to the non‐association groups DX1.25 and 2.5. A significant decrease in P‐FoxO3a (Ser253) expression was observed in all groups compared to the CT group, including the N‐3 group, with no difference between groups.

N‐3 administration did not influence Akt expression during DEXA‐induced atrophy but could significantly affect FoxO3a total expression, showing an independent mechanism that is not Akt dependent.

The mRNA expression of atrogenes (Fig. 4) showed that *MuRF‐1* expression was significantly increased in all groups receiving DEXA in association or not with N‐3 in comparison to the CT group (*P *< 0.05 to DX2.5 and *P *< 0.01 to the other groups). There was a significant difference between the DX2.5+N‐3 group and the DX1.25+N‐3 group (*P *< 0.05).

*Atrogin‐1/MAFbx* expression was significantly increased in all groups receiving DEXA in association or not with N‐3 in comparison to the CT group (*P *< 0.05 to DX2.5 and *P *< 0.001 to the other groups). There was a significant difference on DX2.5+N‐3 compared to the DX2.5 group (*P* < 0.05).

*REDD‐1* expression was significantly increased only the DEXA+N‐3 groups (DX1.25+N‐3 and DX2.5+N‐3 groups) compared to the CT group (*P *< 0.01 and *P *< 0.001, respectively) and showed a tendency to increase in the DX1.25 group in comparison to the CT group (*P *= 0.08). The increase in *REDD‐1* expression was significantly higher in the DX2.5+N‐3 group than in the DX2.5 group (*P *< 0.001).

*REDD2* expression was significantly increased in all groups receiving DEXA in association or not with N‐3 in comparison to the CT group (*P *< 0.01 in the DX1.25 and DX1.25+N‐3 groups and *P *< 0.05 in the DX2.5 and DX2.5+N‐3 groups). There was no difference between groups.

The expression of insulin receptor substrate 1 (*IRS‐1*) was significantly decreased in all study groups receiving DEXA, with a tendency of reduction in the N‐3 group, compared to the CT group (*P *< 0.07 in the N‐3 group and *P *< 0.001 in the other groups, DX2.5+N‐3 *P* < 0.01). On the other hand, the expression of *IRS‐2* was only decreased in the DX1.25+N‐3, DX2.5, and DX2.5+N‐3 groups compared to the CT group (*P *< 0.05 in the DX1.25+N‐3 and DX2.5 groups, and *P *< 0.01 in the DX2.5+N‐3 group) and significantly decreased when comparing DX1.25 and DX2.5 (*P *< 0.05).

DEXA administration leads to increased atrogene mRNA expression in both dosages (DX1.25 and 2.5 mg/kg/day). When associated with DEXA, N‐3 can influence the expression of *REDD‐1* and *Atrogin‐1* at a higher dosage (2.5 mg/kg/day).

### N‐3 supplementation affects muscle PGC1*α* protein expression

**Figure 5:**
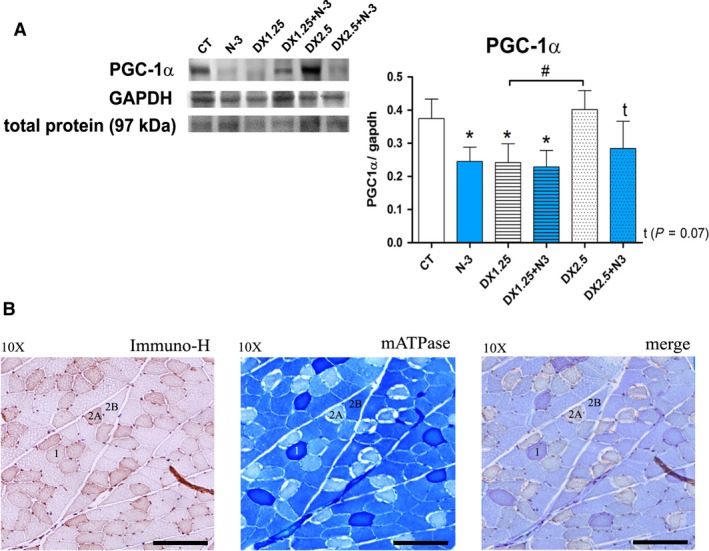
Western blotting analysis of the PGC‐1*α* pathway after 40 days of N‐3 supplementation associated or not with dexamethasone on the last 10 days. (A) Western blotting analysis (*) represents the statistical analysis in comparison to the CT, and (#) represents differences between groups; (B) immunohistochemistry (DAB), mATPase and merge of the same muscle sample area. Student's *t*‐test was used; Legend: * or #*P *< 0.05 (*n* per group: CT **= 8, N3* *= 10, DX1.25* *= 9, DX1.25+N3* *= 7, DX2.5* *= 7, DX2.5+N3* *= 9).

A previous study (Sandri et al. 2006) showed that PGC1*α* is involved in the protection of types 1 and 2A muscle fibers against FoxOs activation in glucocorticoid‐induced muscle atrophy. Considering that we observed increased muscle atrophy in these fiber types after DEXA associated with N‐3, we evaluated PGC1*α* expression in order to verify whether N‐3 could influence its expression. The protein expression of PGC1*α* (Fig. 5A) was significantly decreased in the N‐3, DX1.25, and DX1.25+N3 groups compared to the CT group (*P *> 0.05 for all) and showed a tendency to decrease in the DX2.5+N3 group (*P *= 0.07) and no changes in the DX2.5 group. There was a significant decrease in PGC1*α* expression when comparing the DX1.25 group to the DX2.5 group (*P *< 0.05). Thus, only a low dosage of DEXA (1.25 mg/kg/day) and N‐3 appears to influence muscle PGC1*α* protein expression. Moreover, we confirmed that PGC1*α* was expressed preferentially in types 1 and 2A muscle fibers (Fig. 5B).

### DEXA administration associated or not with N‐3 affects muscle ERK phosphorylation

We aimed to identify whether DEXA in association or not with N‐3 could influence expression of ERK components once the role of the Ras/Raf/MEK/ERK pathway components on glucocorticoid‐induced atrophy is not fully elucidated.

**Figure 6:**
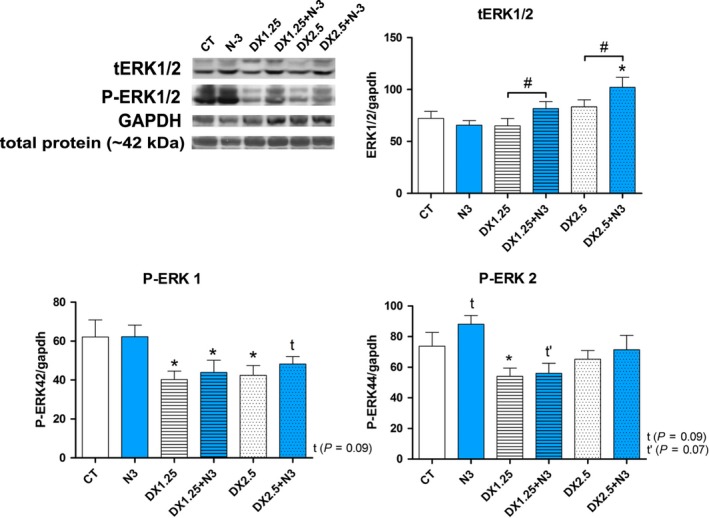
Western blotting analysis of the Ras/Raf/MEK/ERK pathway after 40 days of N‐3 supplementation associated or not with dexamethasone on the last 10 days. (*) represents the statistical analysis in comparison to the CT, and (#) represents differences between groups. Student's *t*‐test was used; Legend: * or #*P *< 0.05 (*n* per group: CT **= 10, N3 = 10, DX1.25 = 9, DX1.25+N3 = 8, DX2.5 = 8, DX2.5+N3 = 9).

Total ERK expression (tERK) was significantly increased only in the DX2.5+N3 group compared to the CT group (*P *< 0.05), showing increased expression compared to the DX2.5 group (*P *< 0.05) (Fig. 6). There was increased expression in the DX1.25+N3 group compared to the DX1.25 group (*P *< 0.05). The phosphorylated form of ERK1 (P‐ERK1) was significantly decreased in the DX1.25, DX2.5, and DX1.25+N3 groups (*P *< 0.05 for all) and showed a tendency to decrease in the DX2.5+N3 group (*P *= 0.09) compared to the CT group. N3 did not influence its expression. P‐ERK2 showed a significant decrease in the DX1.25 group compared to the CT group (*P *< 0.01), and it showed a tendency to increase in the N‐3 group and decrease in the DX1.25+N3 group in relation to the CT group (*P*>0.05).

Therefore, previous and concomitant N‐3 supplementation with DEXA can influence total ERK1/2 expression without changes on its phosphorylated state. DEXA isolated or associated with N‐3 leads to a suppression of P‐ERK1 phosphorylation.

### N‐3 supplementation increases P‐Smad2/3 expression during DEXA‐induced muscle atrophy

Because the myostatin pathway is a negative regulator of muscle trophism and is directly related to glucocorticoid‐induced muscle atrophy (Pereira and Freire de Carvalho 2011), we aimed to evaluate the expression of components of this pathway (Smad2/3 and P‐Smad2/3) after N‐3 and DEXA administration.

**Figure 7:**
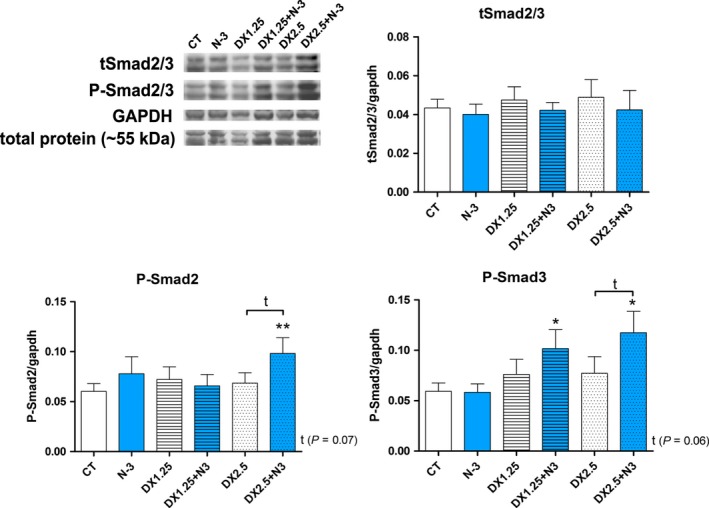
Western blotting analysis of the Myostatin/Smad2/3 pathway after 40 days of N‐3 supplementation associated or not with dexamethasone on the last 10 days. (*) represents the statistical analysis in comparison to the CT, and (#) represents differences between groups. Student's *t*‐test was used; Legend: * or #*P *< 0.05; ** or ##*P *< 0.01 and ***P < 0.001 (*n* per group: CT = 10, N3 = 10, DX1.25 = 9, DX1.25+N3 = 8, DX2.5 = 8, DX2.5+N3 = 8).

Total Smad2/3 expression was unchanged in all groups compared to the CT (Fig. 7 **)**. However, there was a significant increase of P‐Smad2 expression (active form) in the DX2.5+N3 group in relation to the CT group (*P *< 0.01) and a trend of increased expression when comparing the DX2.5+N‐3 to the DX2.5 group (*P *= 0.07).

P‐Smad3 was significantly increased only in the DX+N3 groups compared to the CT group (*P *< 0.05 for both) with a trend of increase in the DX.25+N‐3 group compared with the DX2.5 group (*P *= 0.06). Thus, DEXA administration associated or not with N‐3 does not influence total Smad2/3 protein expression; however, prior and concomitant N‐3 supplementation can lead to an increase of P‐Smad2/3 expression.

### N‐3 supplementation increases autophagic activity during DEXA‐induced muscle atrophy

**Figure 8:**
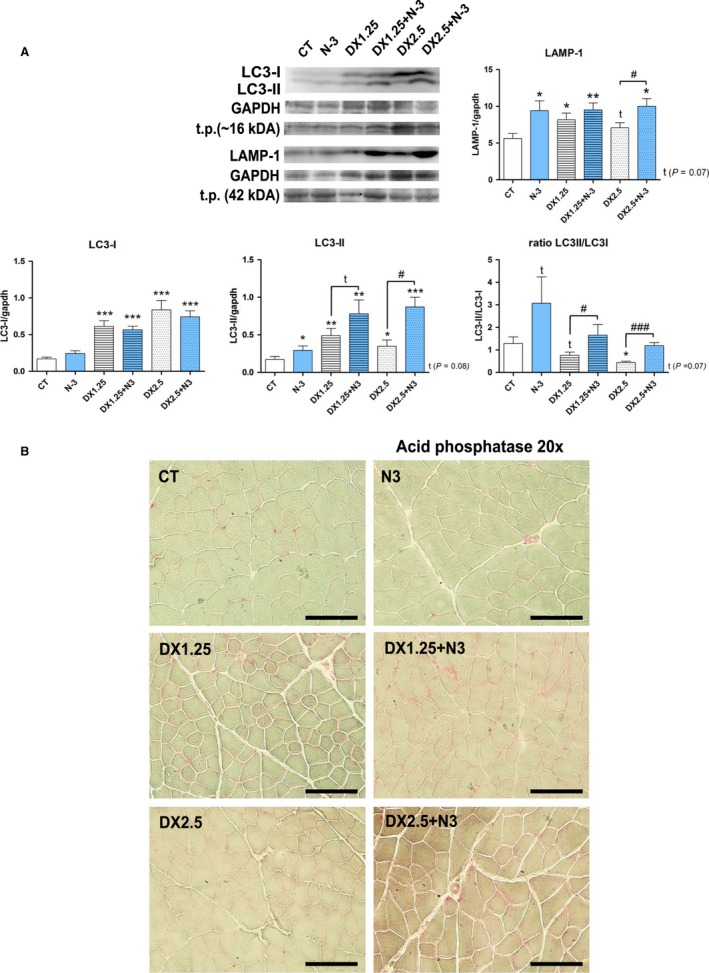
LC3I, LC3II, LC3I/LC3II ratio and LAMP‐1 expressions after 40 days of N‐3 supplementation associated or not with dexamethasone on the last 10 days. (A) Western blotting analysis; (B) Qualitative acid phosphatase reaction in TA muscle slides of all groups; (*) represents the statistical analysis in comparison to the CT, and (#) represents differences between groups. Student's *t*‐test was used; Legend: * or #*P *< 0.05; ** or ##*P *< 0.01 and *** or ###*P *< 0.001; Legend: t.p. = total protein; (*n* per group: CT = 10, N3 = 10, DX1.25 = 8, DX1.25+N3 = 7, DX2.5 = 7, DX2.5+N3 = 9).

The autophagic process is an important part of the muscle atrophy regulation program induced by glucocorticoids (Braun and Marks 2015). Considering the recent high number of works showing enhanced autophagic/lysosomal activity after EPA administration in many tissues other than skeletal muscle, we sought to investigate whether the increased muscle atrophy observed in the association of DEXA with N‐3 was related to activation of the autophagic/lysosomal process. Therefore, LC3B‐I, LC3B‐II, and the LC3II/I ratio as well as LAMP‐1 protein expression were evaluated (Fig. 8A).

LC3‐I expression was significantly increased in all groups receiving DEXA in association or not with N‐3 compared to the CT group (groups DX1.25, DX2.5, DX1.25+N‐3, and DX2.5+N‐3, *P *< 0.001 for all comparisons). LC3‐II expression was increased in all groups compared to the CT group, including the N‐3 group. The DX+N‐3 group showed higher LC3II expression compared to the groups receiving only DEXA: DX1.25 versus DX1.25+N‐3 (*P *= 0.08) and DX2.5 versus DX2.5+N‐3 (*P *< 0.05). The LC3II/LC3I ratio was significantly increased in the DX+N3 groups compared to those receiving only DEXA (DX1.25 versus DX1.25+N‐3 (*P *< 0.05) and DX2.5 versus DX2.5+N‐3 (*P *< 0.001).

A similar observation occurred for LAMP‐1 expression, as there was increased LAMP‐1 expression on the DX1.25, DX1.25+N‐3, and DX2.5 groups. Additionally, the N‐3 group showed increased LAMP‐1 expression in comparison to the CT group.

In addition to the western blotting analysis, we performed a qualitative immunohistochemical analysis to highlight muscular acid phosphatase (Fig. 8B) (lysosomal marker). It showed consistent results to those observed on the western blotting analysis, with remarkable staining of sarcolemmal content on the DX2.5+N‐3 group in comparison to the DX2.5 group.

Based on these findings, we can conclude that DEXA administration significantly increases lysosomal protein expression and that previous and concomitant N‐3 supplementation with DEXA lead to higher autophagic/lysosomal muscle activity compared with DEXA alone, mainly with higher DX dosages.

## Discussion

The ingestion of N‐3 is commonly believed to promote several beneficial effects, but we demonstrate in this study that N‐3 may affect the muscle tissue response to glucocorticoids. N‐3 supplementation potentiated deleterious effects of DEXA on skeletal muscle tissue of Wistar rats when administered concomitantly or previously to DEXA and resulted in greater activation of the UPS and lysosomal degradation systems. This information is clinically relevant, considering the broad use of N‐3 for supplementation in the general population and that glucocorticoids are one of the most prescribed drugs in medical practice, although in inflammatory conditions the administration of both anti inflammatories (DEXA and N‐3) might lead to different outcome other than observed in healthy speciemes.

We previously demonstrated that the association of N‐3 with DEXA also induced greater muscle atrophy in fiber type 1, which is usually more resistant in this model of atrophy (Fappi et al. 2014). Gas chromatography quantifying N‐3 on skeletal muscles by others have always shown the absorption of N‐3 by the skeletal muscle in different models of study after oral supplementation (Andersson et al. 2002; Hess et al. 2012; Rossmeisl et al. 2012). Type 2B muscle fiber atrophy by glucocorticoids is a well‐described side effect (Livingstone et al. 1981) and is believed, in part, to be related to the lower expression of PGC1*α* (a transcription factor related to the mitochondrial biogenesis) in this fiber type (Sandri et al. 2006). Some studies have showed that higher PGC1*α* levels on fiber types 1 and 2A could attenuate FoxOs activity during glucocorticoid‐induced muscle atrophy (Sandri et al. 2006; Qin et al. 2010). Here we demonstrated that PGC1*α* expression was significantly reduced in the skeletal muscle of rats treated with N‐3 alone or in association with DEXA. This finding indicates that the reduction of PGC1*α* levels is a possible mechanism by which N‐3 aggravates glucocorticoid‐induced atrophy in these fiber types. In mice, PGC1‐*α* expression is reduced after administration of DEXA (Jesinkey et al. 2014) and after fish oil supplementation (Martins et al. 2017). On the other hand, some *in vitro* studies have shown that administration of N‐3 increases the expression of PGC1‐*α* in C2C12 cells (Lee et al. 2016) and in C6 cells (Jeng et al. 2009).

PGC1*α* is also associated with muscle‐fiber‐type switch, favoring conversion of fast fibers into slow fibers by interacting with silent information regulator transcript 1 (SIRT1) (Lin et al. 2002) via the AMPK‐SIRT1‐PGC‐1*α* pathway, and chronic AMPK activation induces oxidative myogenic program (Ljubicic et al. 2011). In our study, N‐3 alone or associated with DEXA 1.25 mg/kg/day led to a remarkable decrease in the number of slow (type 1) muscle fibers and an increase in fast (type 2B) fibers. We speculate that the decrease in the percentage of type 1 fibers caused by N‐3 alone or associated to DEXA could be related in part to the AMPK‐SIRT1‐PGC‐1*α*‐pathway.

The IGF‐1 pathway exerts a pivotal role in the maintenance of skeletal muscle trophism (Bodine et al. 2001) through the activity of its central protein Akt (PBK), which directly or indirectly inhibits proteins related to muscle catabolism (i.e., FOXO3) and anabolism (mTOR). In our study, the supplementation of N‐3 with DEXA led to increased expression of total FoxO3a and a reduction in the phosphorylated form of FoxO3a (Ser253) (inactive form) in all groups. These findings indirectly indicate increased ubiquitin‐proteasome system activity, which is confirmed by the high expression of atrogenes, especially in the DX2.5+N‐3 group. Nevertheless, some studies have shown decreased *Atrogin‐1/MAFbx FOXO1* and 4 mRNA expressions after N‐3 administration, in addition to increased *Akt* expression (Liu et al. 2013), Akt and P70S6K protein levels (You et al. 2010).

Studies have shown that DEXA administration (*in vitro*) increases *REDD1* mRNA expression in the first 24 h with no changes of *REDD2* expression (Britto et al. 2014; Wang et al. 2014; Tsuchida et al. 2017). However, after 5 days, *REDD‐2* expression was elevated in rats treated with DEXA without changes in *REDD‐1* (Nishida et al. 2015). These results corroborate our results on *REDD1/2* expression regarding DEXA administration (without N‐3 supplemented), which showed a REDD1/2 time‐dependent action on glucocorticoid‐induced muscle atrophy. However, when associated to N‐3, *REDD‐1* expression remained elevated even after 10 days of DEXA administration, which points to a prolonged state of muscular atrophy.

Considering the impact of glucocorticoids on FOXO nuclear translocation, the higher *Atrogin‐1/MAFbx* and *MURF‐1* expression in groups receiving only DEXA was expected; however, animals from the DX2.5+N‐3 group presented higher expression of *Atrogin‐1*/*MAFbx* than those from DX2.5, which is in accordance with our previous findings (Fappi et al. 2014) observed when a higher dosage of DEXA (5 mg/kg/day) was used. Although the mechanisms by which N‐3 can influence the expression of REDD 1/2 and *Atrogin‐1/MAFbx* when associated with DEXA are still unknown, they may be related to the elevated FOXO3a and P‐SMAD2/3 protein expression observed in our study.

Insulin and IGF1 receptors are tyrosine kinase proteins that phosphorylate various proteins through a signaling cascade, including the insulin receptor substrates IRS‐1 and IRS‐2, which have a key role in muscle homeostasis, glucose metabolism, activating Akt/mTOR signaling, and limiting AMPK activation (Long et al. 2011). We found that groups treated only with DEXA had reductions in the *IRS‐1/2* gene, whereas N‐3 supplementation alone reduced the expression of *IRS‐1*. In specific conditions, such as diabetes, N‐3 can increase *IRS‐1* gene expression (Hu et al. 2014). In non‐pathologic conditions, as demonstrated by Taouis et al. (2002) in rats receiving an N‐3 supplementation, despite a significant decrease in *P85‐α* (PI‐3K substrate) mRNA expression, *IRS‐1* expression remained at normal response.

The Ras/Raf/MEK/ERK pathway is related to the muscle mass regulation in addition to other cell functions including the proliferation, differentiation, apoptosis, and survival of competent mitotic cells (Kolch 2000; Glass 2003). Zheng et al. (2010) showed in L6 cells that active form of FOXO3a increases IRS‐2 in the skeletal muscle, inducing greater MEK/ERK pathway and Sp1 activation and consequent activation of ubiquitin (UbC), leading to muscular atrophy. In contrast, in our study, DEXA administration led to a significant reduction in ERK phosphorylation. Peng et al. (2012) have shown in C2C12 cells that N‐3 (EPA/DHA) may affect myoblast proliferation by inhibiting ERK phosphorylation. However, the MEK/ERK pathway seems to have distinct roles depending on muscle maturity (Rommel et al. 1999). For example, Salto et al. (2014) showed that ERK activation (by sodium tungstate) in L6 cells is capable of increasing protein synthesis and decreasing protein degradation by acting on FoxO3a and mTOR expression. In addition, ERK activation improves protein turnover in DEXA‐treated skeletal muscle cells (Salto et al. 2014). Corroborating this information, Marzuca‐Nassr et al. (2016) conducted limb suspension experiments in rats that showed a significant decrease in P‐ERK1/2, showing that muscle atrophy leads to MEK/ERK pathway inhibition; in addition, EPA does not influences ERk1/2 expression, what points to a specific N‐3 influence in different muscle atrophy model.

In contrast to the trophic effects of the IGF‐1 pathway, the Myostatin/Smad2/3 pathway is related to negative regulation of muscle mass through activation of Smad proteins, which leads to inhibition of muscle growth and activation of the ubiquitin‐proteasome degradation system (Wang et al. 2016).

We observed that the DX2.5+N‐3 group had higher P‐Smad2/3 and that N‐3 alone did not influence SMAD/P‐SMAD signalization, even though decreases in P‐SMAD2/3 phosphorylation after N‐3 supplementation have previously been reported by others (Chen et al. 2012; Ventro et al. 2017).

Glucocorticoid‐induced muscle atrophy is related to different catabolic systems, including the autophagic/lysosomal activation (Hasselgren 1999; Schakman et al. 2013). One of the main mechanisms of lysosomal activation by glucocorticoids is the inhibition of IGF‐1/PI‐3K/Akt/mTOR pathway signaling. Thus, we analyzed the expression of some components of this system in our model of DEXA‐induced atrophy.

Our observations showed that DEXA caused increased LC3‐I, LC3‐II, and LAMP‐1 muscle expression, mainly in relation to the lowest dosage administered (DX1.25), with a trend in normalization by the DX2.5 dosage. The association with N‐3 significantly increased LC3‐II expression and the LC3II/LC3‐I ratio. These findings confirm the effect of DEXA on the lysosomal system as well as the potential influence of N‐3 on this pathway in muscle tissue.

The autophagic process in glucocorticoid muscle atrophy is well described. Giron et al. (2015) administered DEXA (0.1 mg/kg/day) for 21 days *in vivo* (Sprague Dawley rats), leding to an increase in LC3‐II and P62 protein expression in skeletal muscle, which were attenuated by prior and concomitant administration of *β*‐Hydroxy‐*β*‐methylbutyrate (HMB ‐ Leucine metabolite) through mechanisms involving activation of Akt and inhibition of FOXO3a (Giron et al. 2015). Similarly increased expression of autophagic components has been shown *in vitro* (Hudson et al. 2014; Troncoso et al. 2014) in association with a potential role of glucocorticoid on AMPK‐dependent mitochondrial clearance (Troncoso et al. 2014).

Recently, many studies have shown that N‐3 has the property of enhancing autophagy in several models, such as glial tumor cells (D54MG, U87MG, U251MG, and GL261) (Kim et al. 2018), renal tissue (ischemia‐reperfusion study) (Gwon et al. 2017), immune system/macrophages (Shen et al. 2017), central nervous systems (CNS)/microglia (Inoue et al. 2017), ocular cells/ARPE‐19 (Koskela et al. 2016), CNS/Purkinje cells (Bak et al. 2015), pulmonary/A549 cells (Yao et al. 2015), and bone marrow/mBMMSC cells (Gao et al. 2016). In this last study, the authors demonstrated that DEXA with EPA association increases cellular autophagy compared to DEXA alone. According to the authors, the modulation of autophagy by DEXA and N‐3 occurred through GPR120, AMPK, and mTOR activation.

In conclusion, our study shows that N‐3 supplementation can act on regulation of muscle mass and significantly aggravate dexamethasone‐induced muscle atrophy in rats by increasing UPS and autophagic/lysosomal system activation, and decreasing muscle PGC1‐alpha protein expression. Despite the known healthy effects of fish oil (EPA/DHA) for many organs and systems or even pathologic/inflammatory conditions, there is a potential role of EPA in regulation of the autophagic/lysosomal process, which can be beneficial to some tissues/conditions but harmful to others like the glucocorticoid‐induced muscle atrophy.

## Conflict of Interest

The authors declare no conflict of interest.
